# Editorial: Addressing equity issues in traffic injury prevention

**DOI:** 10.3389/fpubh.2024.1465197

**Published:** 2024-08-12

**Authors:** Jingwen Hu, Jessica Jermakian, Johan Iraeus, Jonathan Rupp

**Affiliations:** ^1^Transportation Research Institute, University of Michigan, Ann Arbor, MI, United States; ^2^Vehicle Research, Insurance Institute for Highway Safety (IIHS), Arlington, VA, United States; ^3^Department of Mechanics and Maritime Sciences, Chalmers University of Technology, Gothenburg, Sweden; ^4^School of Medicine, Emory University, Atlanta, GA, United States

**Keywords:** equity, traffic injury prevention, human body model (HBM), field data analysis, human diversity

Recent literature has shown that there is a growing concern of safety equity in motor-vehicle crashes (MVCs), but studies for addressing this issue are limited. Field data have shown that different demographic, socioeconomic, or geographic groups of people may have significantly different injury risks in MVCs. Increased traffic fatalities or serious injuries have been reported in older adults, people with obesity, and female occupants, as well as in low-income communities, although the causes of these issues may vary widely. Unfortunately, safety equity will likely get worse without action. The projected increase of older and obese populations in many countries and their interacting effects on sex, income and education further motivate future efforts to develop more advanced safety designs, standards and public policies for improving safety equity and mitigating injuries in these vulnerable populations.

The goal of this Frontiers' Research Topic “*Addressing equity issues in traffic injury prevention*” is to identify, investigate, and address safety equity issues among traffic injury prevention through multidisciplinary research studies. The accepted research articles include a study on field data analysis, and three studies on computational modeling and their applications for addressing safety equity.

Cronn et al. analyzed clinical data from a large trauma database to identify sex-related difference of injury patterns in MVCs. It was found that female occupants tend to have higher severity of pelvis and liver injuries than male occupants, which may be related to how their bodies interact with safety equipment. This study validated sex-related safety equity concerns presented in the previous studies.

Sun et al. presented a study on developing adaptive restraint systems for a diverse population through population-based crash simulations and machine-learning algorithms. Occupant sex, stature, and body mass index (BMI) were considered in the crash simulations, and an optimization framework accounting for occupant covariates and the associated injury risk uncertainties were developed to seek an optimal restraint design policy that can adapt to occupant characteristics and minimize the population injury risks as a whole. This study demonstrated the potential for adaptive restraint systems to effectively reduce the injury variations and improve safety equity among the population.

Leo et al. developed an integrated virtual assessment framework considering both male and female pedestrians to evaluate injury risks. A total of 61,914 virtual testing scenarios were derived from real-world pedestrian crash scenarios. The effectiveness of Autonomous Emergency Braking (AEB) and the injury risk differences between male and female pedestrians were investigated. It was reported that an 81.7% reduction in injury risk was achieved by implementing an AEB for pedestrians. The findings highlighted the differences in injury risks between male and female pedestrians and the effectiveness of active and passive safety technologies on pedestrian protection.

Corrales et al. reported a study focusing on the mechanisms of why older populations sustained higher thoracic injury risks in MVCs. Finite element human body models were developed for young and aged occupants by integrating a biofidelic cortical bone constitutive model and population-based bone material properties. They found that shear stress led to an increased number of rib factures in the aged human model during side impacts, which is not correlated to chest compression, a commonly used thorax injury measure. The findings of this study will benefit assessment and design of future safety systems that better account for thoracic injuries in older populations.

This Research Topic includes four studies that address a wide range of safety equity issues related to sex, age, and obesity levels in MVCs. The inability of current vehicle safety designs to deal with human variation is a direct contributor to those issues. Nearly all current vehicle safety design optimizations use crash test dummies, also known as anthropomorphic test devices (ATDs). That is, the design optimization is performed by changing the safety system variables to improve injury-related metrics, such as forces, deflections, and accelerations, obtained from ATDs. Importantly, ATDs represent only a small number of occupant sizes and shapes. For example, adults are represented by three sizes (so-called 5th-percentile female, 50th-percentile male, and 95th-percentile male). Only the first two are used in regulatory and consumer information testing, so in practice the entirety of the variability in the adult population is represented by two sizes of ATDs. Therefore, they do not consider age, sex, and obesity effects on the geometrical and biomechanical variations among the population. As a result, these vulnerable populations and the associated large variations in impact responses and injury tolerances are not directly represented in the current safety evaluation process.

This Research Topic also highlights the importance of computational modeling for addressing the safety equity issues, including developing computational human body models for representing occupants in different ages, developing pedestrian injury assessment framework by integrating both male and female human body models, and applying crash simulations and machine-learning algorithms to optimize smart safety technologies that can adapt to the diverse population.

In conclusion, we hope that this Research Topic plays a role in advancing the understanding of equity issues in traffic injury prevention and provides examples of solutions. However, the presented works offer only a glimpse of the current progress for addressing such a complex problem. Many important Research Topics require further investigation, including but not limited to, improving usage rate of seat belt and child restraints for different socioeconomic groups, better understanding human variability on impact responses and tolerance, developing and validating parametric human modeling for representing the diverse population, developing automated virtual testing framework for future vehicle safety assessment, and field data fusion and analysis to better quantify variations in population characteristics and their associated effects on traffic injuries. We truly believe that the goal of equity in traffic injury prevention is achievable through strategic investment and dedication from the safety community, which will ensure “Safety for All.”

